# Autopsy after transcatheter aortic valve implantation

**DOI:** 10.1007/s00428-017-2076-4

**Published:** 2017-01-27

**Authors:** F. van Kesteren, E. M.A. Wiegerinck, S. Rizzo, J. Baan, R.N. Planken, J. H. von der Thüsen, H.W.M. Niessen, M.F.M. van Oosterhout, A. Pucci, G. Thiene, C. Basso, M.N. Sheppard, K. Wassilew, A. C. van der Wal

**Affiliations:** 1 Heart Centre, Academic Medical Centre, Amsterdam, The Netherlands; 2 Department of Radiology, Academic Medical Centre, Amsterdam, The Netherlands; 3 Department of Cardiac, Thoracic, and Vascular Sciences, Cardiovascular Pathology Unit, University of Padua, Padua, Italy; 4 Department of Pathology, Erasmus Medical Centre, University of Rotterdam, Rotterdam, The Netherlands; 5 Department of Pathology and Cardiac Surgery, ICaR-VU, VU Medical Centre, Amsterdam, The Netherlands; 6 Department of Pathology, Sint Antonius Hospital, Nieuwegein, The Netherlands; 7 Department of Pathology, Pisa University Hospital, Pisa, Italy; 8 Department of Pathology, St George Medical School, University of London, London, UK; 9 Department of Cardiothoracic and Vascular Surgery, Cardiac Pathology Unit, Deutsches Herzzentrum Berlin, Berlin, Germany; 10 Department of Pathology, Rigshospitalet, Copenhagen, Denmark; 11 Department of Pathology, Academic Medical Centre–University of Amsterdam, Meibergdreef 9, 1105 AZ Amsterdam, The Netherlands

**Keywords:** TAVI, TAVR, Cause of death, Pathology, Autopsy

## Abstract

Autopsy after transcatheter aortic valve implantation (TAVI) is a new field of interest in cardiovascular pathology. To identify the cause of death, it is important to be familiar with specific findings related to the time interval between the procedure and death. We aimed to provide an overview of the autopsy findings in patients with TAVI in their medical history divided by the timing of death with specific interest in the added value of autopsy over a solely clinically determined cause of death. In 8 European centres, 72 cases with autopsy reports were available. Autopsies were divided according to the time interval of death and reports were analysed. In 32 patients who died ≤72 h postprocedure, mortality resulted from cardiogenic or haemorrhagic shock in 62.5 and 34.4%, respectively. In 31 patients with mortality >72 h to ≤30 days, cardiogenic shock was the cause of death in 51.6% followed by sepsis (22.6%) and respiratory failure (9.7%). Of the nine patients with death >30 days, 88.9% died of sepsis, caused by infective endocarditis in half of them. At total of 12 patients revealed cerebrovascular complications. Autopsy revealed unexpected findings in 61.1% and resulted in a partly or completely different cause of death as was clinically determined. Autopsy on patients who underwent TAVI reveals specific patterns of cardiovascular pathology that clearly relate to the time interval between TAVI and death and significantly adds to the clinical diagnosis. Our data support the role of autopsy including investigation of the cerebrum in the quickly evolving era of cardiac device technology.

## Introduction

Aortic valve stenosis is the most common valvular heart disease in adults and has a strong age associated incidence [4]. As a result of increasing life expectancies, the prevalence of the disease increases simultaneously [3]. For patients considered to be at high or prohibitive risk for conventional cardiac surgery, transcatheter aortic valve implantation (TAVI) evolved as an alternative, less invasive treatment [1, 7, 12]. In TAVI, no sternotomy is necessary and the aortic valve is not excised; instead, a catheter is used to implant a bioprosthetic valve over the native valve. Over the past decade, the increased prevalence of aortic valve stenosis combined with improvements in material, experience and techniques resulted in an expanding number of TAVI procedures.

The TAVI population is relatively old and has complex comorbidities inhibiting conventional surgery. The reported 1-year mortality after TAVI ranges from 14 to 31% [1, 9, 18], and the novelty of the procedure creates a new field of interest in cardiovascular autopsy pathology. Since the number of patients treated with TAVI increases, a rise in submissions in autopsy practice is expected. However, current literature reviewing the causes of death at autopsy after TAVI is scarce and consists of small single-centre case series [8, 9]. Moreover, clinicians may waive the option of autopsy in this elderly population since it is not known what the added value of autopsy after TAVI is. To identify the cause of death in this population with significant comorbidities, it is important to be familiar with the specific procedure-related findings at autopsy. We hypothesise that the findings at autopsy after TAVI will differ substantially according to the time interval of death after the procedure. Therefore, we provide an overview of autopsy findings after TAVI procedures in multiple centres with specific expertise in cardiovascular pathology and emphasise the timing of death after the procedure. In addition, we consider the added value of an autopsy for determination of the cause of death by comparing the pathologic findings at autopsy with the clinically determined cause of death.

## Methods

For this observational retrospective study, institutional autopsy registries of eight European pathology laboratories with specific cardiovascular expertise were screened for patients with TAVI in their medical history. Registries of the following laboratories were screened: Academic Medical Centre Amsterdam, VU Medical Centre Amsterdam, Erasmus Medical Centre Rotterdam, Sint Antonius Nieuwegein, Deutsches Herzzentrum Berlin, St. George Hospital Medical School London, Cardiovascular Pathology Unit University of Padua and the Pisa University Hospital. Autopsies were performed according to local legislation. All available autopsy reports of these hospitals were included in our analysis. A standardised questionnaire was used to evaluate the autopsy reports and archived tissues (heart specimens and/or paraffin blocks). The following information was required for the questionnaire: baseline and procedural characteristics, the clinically suspected cause of death and the primary cause of death found at autopsy, specific findings of cardiac autopsy (cardiac hypertrophy and/or dilatation, native valve abnormalities, pathology of the aortic root, signs of ischemic heart disease, positioning of the implanted prosthesis, signs of valvular thrombosis or endocarditis, procedure-related trauma) and findings at cerebral autopsy.

In order to divide the autopsies in time intervals of death, we used the standardised endpoints of the VARC-2 recommendations [8]. We distinguished cases with immediate procedural mortality (≤72 h postprocedure), procedural mortality (>72 h to ≤30 days postprocedure) and postprocedural mortality (>30 days postprocedure).

## Results

In the 8 participating centres, 72 cases of autopsy after TAVI were available. Procedures were performed between 2007 and 2015 with the self-expandable Medtronic CoreValve (Medtronic Inc., Minneapolis, MN, USA), the balloon expandable Edwards SAPIEN/SAPIEN XT/Sapien 3 (Edwards Life Sciences LCC, Irvine, CA, USA) or the Lotus (Boston Scientific Corporation, Marlborough, MA, USA) bioprostheses. Reported access routes were transfemoral (*n* = 29), transapical (*n* = 28), transaortic (*n* = 5) and subclavian (*n* = 2) (not documented, *n* = 8). Eleven procedures were performed under conscious sedation with local analgesia (15.3%), all other under general anaesthesia. In seven patients, the performed TAVI was superimposed on a previously surgically inserted degenerated aortic valve prosthesis (*n* = 6) or a not correctly positioned transcatheter prosthesis (*n* = 1) (‘valve-in-valve procedures’, 9.7%). Baseline characteristics of all patients divided per time interval of death and general findings at autopsy are presented in Table 1. In all but one case (98.6%), a pre-existent damaged heart was found, with hypertrophy and/or dilatation and scarring fibrosis due to previous infarctions. In 52 patients, 72.2% cerebral autopsy was performed in addition to the cardiac autopsy.

**Table 1:** Baseline characteristics and general autopsy findings at time of autopsy per time interval of death

Characteristics	≤72 h, *n* = 32	>72 h to ≤30 days, *n* = 31	>30 days, *n* = 9
Female	19 (59.4%)	16 (51.6%)	3 (33.3%)
Age	80 (±5)	79 (±8)	82 (±8)
Body mass index	27.2 (±8.9)	25.5 (±13.6)	29.0 (±4.9)
Medical history			
Hypertension	24 (75.0%)	19 (61.3%)	7 (77.8%)
Hypercholesterolemia	14 (43.8%)	12 (38.7%)	6 (66.7%)
Diabetes	16 (50.0%)	11 (35.5%)	1 (11.1%)
Atrial fibrillation	12 (37.5%)	15 (48.4%)	5 (55.6%)
Stroke	2 (6.2%)	6 (19.4%)	0 (0.0%)
CABG	13 (40.6%)	9 (29.0%)	3 (33.3%)
PCI	12 (37.5%)	11 (35.5%)	1 (11.1%)
Heart weight	584.9 (±148.0)	569.9 (±116.3)	602.4 (±196.6)
Cardiac hypertrophy	28 (87.5%)	28 (90.3%)	9 (100%)
Cardiac dilatation	14 (43.8%)	12 (38.7%)	5 (55.6%)
Old myocardial infarction	22 (68.8%)	18 (58.1%)	4 (44.4%)
Recent myocardial infarction	12 (37.5%)	12 (38.7%)	2 (22.2%)
Procedure-related haemorrhage	11 (34.4%)	2 (6.5%)	–
Not correctly positioned prosthesis	1 (3.1%)	5 (16.1%)	1 (11.1%)
Thrombus at prosthesis site	1 (3.1%)	3 (9.7%)	1 (11.1%)
Cerebrovascular accident	3 (9.4%)	7 (22.6%)	2 (22.2%)
Ischemic	3 (9.4%)	6 (19.4%)	2 (22.2%)
Haemorrhage	2 (6.3%)	3 (9.7%)	–
Microbleeds	1 (3.1%)	4 (12.9%)	–
Endocarditis	– (0.0%)	1 (3.2%)	4 (44.4%)
Sepsis	– (0.0%)	8 (25.8%)	8 (88.9%)

Dichotomous data are presented as number of patients (%); continuous data are expressed as mean (±SD)

*CABG* coronary artery bypass graft, *PCI* percutaneous coronary intervention

### Immediate procedural mortality (≤72 h postprocedure)

A total of 32 patients died within 72 h of the procedure (44.4% of the total cohort) of whom 19 patients died on the day of the TAVI. In three patients, no prosthesis was implanted since death occurred in an early phase of the procedure. The clinically suspected causes of death are described in Table 2. Death due to cardiogenic shock was clinically suspected in most patients, 22 of the 32 (68.8%). The primary causes of death found at autopsy are described in Table 3. In 20 of the 32 patients (62.5%), the primary cause of death was a cardiogenic shock due to cardiac hypertrophy and dilatation (end-stage cardiac failure), within addition signs of acute ischemia in 10 of these 20 patients. In these cases, the procedure was interpreted as the eliciting factor of death. In one patient, a large hematoma in the right atrial wall was found; histological examination revealed recent bleeding in proximity to the AV node and myocardial ischemia. Haemorrhagic shock was observed as the primary cause of death in 11 patients (34.4%), mostly as a consequence of aortic annulus rupture. Although brain autopsy revealed signs of a cerebrovascular accident in three patients (Table 1), in only one patient, the primary cause of death was described to be due to an ischemic stroke caused by thromboembolic occlusion of the basilar artery. Histological analysis revealed that the thrombotic material originated from a thrombus mass at the prosthesis site. Infectious disease was not observed in patients who died within 72 h postprocedure (Tables 1 and 4).

**Table 2:** Clinical suspected causes of death per time interval

Cause of death	≤72 h, *n* = 32	>72 h to ≤30 days, *n* = 31	>30 days, *n* = 9
Cardiogenic shock	22 (68.8%)	14 (45.2%)	0
Electromechanical dissociation	9	1	–
Acute myocardial infarction	5	2	–
Acute worsening heart failure	3	8	–
Unknown cause	5	3	–
Hemorrhagic shock	7 (21.9%)	4 (12.9%)	0
Annular rupture	2	–	–
Retroperitoneal hematoma	1	1	–
Pericardial perforation	1	–	–
Gastrointestinal	–	2	–
Unknown origin	3	1	–
Sepsis	0	6 (19.4%)	7 (77.8%)
Origin endocarditis	–	1	1
Origin infected feet	–	–	1
Origin pneumonia	–		2
Origin cellulitis	–		1
Sepsis unknown origin	–	5	1
Respiratory failure	1 (3.1%)	2 (6.5%)	0
Cerebral infarction	2 (6.3%)	3 (9.7%)	0
Basilar artery	2	1	–
Left middle cerebral artery	–	1	–
Diffuse	–	1	–
Multiorgan failure unknown origin	0	1 (3.2%)	0
Renal insufficiency	0	1 (3.2%)	0
Malignancy	0	0	1 (11.1%)
Unknown cause	0	0	1 (11.1%)

**Table 3:** Primary causes of death at autopsy per time interval of death

Cause of death	≤72 h, *n* = 32	>72 h to ≤30 days, *n* = 31	>30 days, *n* = 9
Cardiogenic shock	20 (62.5%)	16 (51.6%)	1 (11.1%)
End-stage cardiac failure with recent ischemia	10	6	1
End-stage cardiac failure without recent ischemia	9	7	–
Large hematoma AV node damaging conduction system with recent ischemia	1	–	–
Mechanical obstruction mitral valve by TAVI prosthesis	–	2	–
Right ventricular failure secondary to pulmonary embolisms	–	1	–
Hemorrhagic shock	11 (34.4%)	2 (6.5%)	0
Annular rupture	5	–	–
Retroperitoneal hematoma	1	1	–
Perforated pericardium by pacemaker wire in compromised heart	1	–	–
Dissection thoracic aorta	1	–	–
Anastomosis previous CABG	1	–	–
Transmural tear aortic wall	1	–	–
DIC	1	1	–
Sepsis	0	7 (22.6%)	8 (88.9%)
Cardiac origin endocarditis of TAVI prosthesis	–	–	4
Cardiac origin pacemaker wire	–	1	–
Not (directly) cardiac origin pneumonia	–	3	2
Not (directly) cardiac origin ischemic colitis	–	1	1
Not (directly) cardiac origin necrotizing cholecystitis	–	1	–
Not (directly) cardiac origin unknown, candida found	–	1	–
Not (directly) cardiac origin cellulitis	–		1
Respiratory failure—ARDS	0	3 (9.7%)	0
Cerebral infarction	1 (3.1%)	2 (6.5%)	0
Basilar artery	1	1	–
Left middle cerebral artery	–	1	–
Cerebral edema	0	1 (3.2%)	0

*ARDS* acute respiratory distress syndrome, *CABG* coronary artery bypass graft, *DIC* diffuse intravascular coagulation

### Procedural mortality (>72 h to ≤30 days postprocedure)

Thirty-one patients died between 72 h and 30 days after the procedure (43.1% of the total cohort) after a mean of 12 days after TAVI (Table 1). The clinically suspected cause of death was cardiogenic in 14 patients (45.2%), followed by sepsis in 6 patients (19.4%) (Table 2). The primary causes of death found at autopsy are described in Table 3. The leading cause was of cardiogenic origin in 16 patients (51.6%). Thirteen patients died due to cardiac hypertrophy and dilatation (end-stage cardiac failure), with or without signs of acute myocardial ischemia. In one of the patients with ischemia, the prosthesis was implanted too high, obstructing the coronary ostia. In addition, two patients had worsening of heart failure due to severe mitral regurgitation caused by obstruction of the mitral leaflets by a too low implanted prosthesis. In another patient, the prosthesis pressed on the cardiac septum, damaging the conduction system. Two patients died of haemorrhagic shock (6.5%) and seven of sepsis with multiorgan failure (22.6%). In two of the patients with sepsis, thrombotic material was found at the site of implantation (one patient with candida sepsis whom had multiple abscesses in the myocardium and one patient with ischemic colitis). Acute respiratory distress syndrome (ARDS) leading to respiratory failure was the cause of death in three patients (9.7%). Although signs of a cerebrovascular accident were found in seven patients (Table 1), only in two patients a cerebral infarct was described as the primary cause of death (6.5%) (Table 3). One of these patients also had extensive infectious endocarditis of the TAVI prosthesis with thrombus at the prosthesis site and myocarditis. In one patient with a low inserted prosthesis, cerebral oedema with herniation of the cerebellar tonsils was interpreted as primary cause of death.

### Postprocedural mortality (>30 days postprocedure)

Nine patients died more than 30 days after TAVI (12.5%). The time interval between TAVI and onset of death ranged from 33 to 936 days. Septic shock was the most frequent clinically suspected cause of death (Table 2). Sepsis was also the leading cause of death at autopsy (the major pathological finding in eight patients; Table 3). In four patients, sepsis was a consequence of infective endocarditis; two of them died within 2 months and two after 2 years. In a patient who died of sepsis caused by pneumonia, a too low implanted prosthesis was found, obstructing the mitral valve but not directly leading to death. Although not defined as the main cause of death, in two patients, signs of a cerebrovascular accident were found (Table 1). These cerebral infarcts originated from septic embolisms after endocarditis and ischemic colitis, respectively.

### Correlation of clinically suspected cause of death and cause of death at autopsy

In 28 of the 72 patients, the clinically determined cause of death was confirmed as the primary cause of death at autopsy (38.9%). In 44 patients, autopsy revealed relevant additional findings (61.1%) that resulted in a partly or completely different cause of death as was determined clinically (34.7 and 26.4%, respectively). In patients with mortality between 72 h and 30 days, the discrepancy between the clinically suspected cause of death and the cause of death at autopsy was highest as the clinically suspected cause was only confirmed in 9 of the 31 patients (29.0%). The correlation per category of the suspected cause of death is described in Table 4.

**Table 4:** Correlation clinical suspected cause of death (COD) and cause of death at autopsy

No. of patients per category clinical suspected COD	COD autopsy completely same as clinically suspected	COD autopsy partly same as clinically suspected	COD autopsy completely differ from clinically suspected
Cardiogenic shock, *n* = 36	17 (47.2%)	12 (33.3%)	7 (19.4%)
Hemorrhagic shock, *n* = 11	5 (45.5%)	4 (36.4%)	2 (18.2%)
Sepsis, *n* = 13	3 (23.1%)	6 (46.2%)	4 (30.8%)
Respiratory failure, *n* = 3	2 (66.7%)	–	1 (33.3%)
Cerebral infarction, *n* = 5	1 (20.0%)	3 (60.0%)	1 (20.0%)
Multiorgan failure, *n* = 1	–	–	1 (100%)
Renal insufficiency, *n* = 1	–	–	1 (100%)
Malignancy, *n* = 1	–	–	1 (100%)
Unknown cause, *n* = 1	–	–	1 (100%)

## Discussion

With an expanding number of TAVI procedures worldwide, pathologists will be exposed more often to autopsies of patients with TAVI in their medical history. This study is the largest and only international multicentre study on autopsy findings in this relatively new patient population. We found different patterns of the causes of death amongst the different time intervals between the procedure and death and also demonstrated a substantial additional value of autopsy to the solely clinical determined cause of death. Previous reports on autopsy after TAVI are limited to small single-centre analysis of autopsies in 13 or 17 cases and did not focus on discrepancies between clinically determined causes of death and the subsequently derived findings at autopsy [10, 15].

### Cardiac pathology in relation to the time interval after TAVI

TAVI is the preferred therapy for patients with high or prohibitive risk for surgery. TAVI patients are relatively old and have an extensive medical history, the pathologist should be aware of. This was also seen in our population, with a mean age of 80 years and a high incidence of co-morbidities including cardiovascular risk factors and an overrepresentation of previous coronary procedures. As could be expected in patients with (a history of) aortic valve stenosis, findings of cardiac hypertrophy and dilatation and evidence of old ischemia were noted at autopsy in nearly all patients.

It appeared of pivotal importance to aim attention at the time interval between the TAVI procedure and death. Cardiogenic and especially haemorrhagic shock occurred less frequently as time after the procedure passed. On the contrary, respiratory failure and sepsis only occurred after 72 h. Amongst patients who died after 72 h, a high incidence of prosthetic valve endocarditis of 12.5% (5 of the 40 cases) was present, which was even higher than the reported incidence in literature of 3.1% in the first year after TAVI [16]. Furthermore, we demonstrated the importance of paying attention to the position of the prosthesis at autopsy. Incorrect positioned prosthesis could be related to conduction disorders, severe mitral regurgitation or coronary obstruction [5, 11, 17]. The demonstration of damage of the conduction system by histopathological analyses is difficult. In most patients, in this study, a thorough examination of the conduction system was not described. We suppose that systematic histopathological investigation of the AV node and proximal bundle branches would reveal a much higher incidence of haemorrhagic and or ischemic changes at these specific sites.

### TAVI and cerebral pathology

Cerebral infarcts are a well-known complication of TAVI with a reported 30-day clinical stroke incidence of approximately 3–4% and the highest incidence within 24 h after the procedure [2, 6, 14]. We found old and new ischemic or haemorrhagic lesions in 12 of the 52 patients in whom cerebral autopsy was performed. This implies that, although not always considered the primary cause of death, unexpected cerebral lesions are found in a large proportion of the TAVI population. Moreover, in two patients with a suspected cerebral infarct, no ischemic or haemorrhagic brain lesions were found. Therefore, cerebral autopsy can be considered extremely helpful in determining the cause of death after TAVI, and clinicians should be encouraged to ask for permission of the relatives for this type of autopsy.

A striking finding in our autopsies was the detection of thrombus at the site of the prosthesis in five patients (6.9%). Recently, Makkar et al. reported a high incidence of 13 to 40% of reduced leaflet motion after implantation of a bioprosthetic valve, identified on computed tomography (CT). This condition resolved with administration of therapeutic anticoagulation, suggesting that reduced leaflet motion could be associated with subclinical leaflet thrombosis [13]. In agreement with the report of Makkar et al., we hypothesise that although stroke after TAVI is multifactorial, valve thrombus with possible embolisation of thrombotic material may play an important role in developing cerebral ischemic lesions. In an earlier reported series of thromboembolic events after TAVI, we demonstrated the importance of removal of the prosthesis from its location in the left ventricular outflow tract for inspection on the presence of thrombus [20]. This is further supported by the analysis in this current study; in three of the five patients with prosthesis site thrombus, signs of ischemic stroke were found.

### Clinical diagnosis versus diagnosis at autopsy

We demonstrated that clinically relevant extra findings were found at autopsy in 61.1% of the patients, which completely changed the perception on the cause of death in 26.4% of all patients. These large discrepancies highlight the vital importance of autopsy over a solely clinically determined cause of death, not just in the immediate postprocedural period but also later after TAVI. In addition to this benefit of autopsy in determining the causes of death, Vogel et al. recently demonstrated a possible added value of performing postmortem computed tomography (angiography) (PMCT) after TAVI [19]. The authors were able to show coronary artery disease and position of the implanted device and demonstrated the specific localisation of haemorrhage [19]. PMCT with angiography could be considered valuable in addition to autopsy to identify the cause of death after TAVI.

### Recommendations for autopsy

**Fig. 1:**
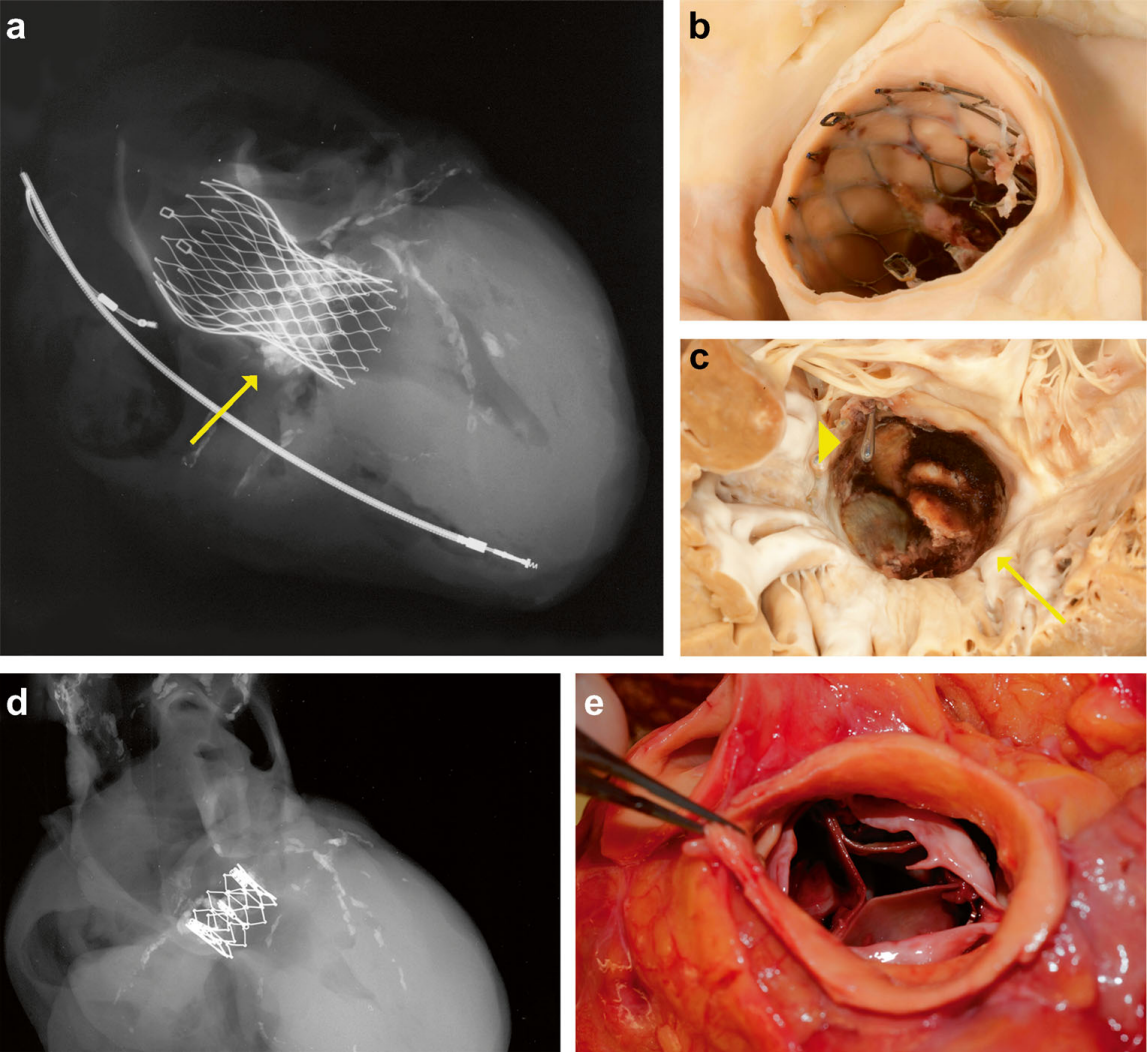
Examples of two different types of TAVI prosthesis. **a** Postmortem X-ray of the heart showing the result of TAVI with CoreValve prosthesis in aortic root position. Calcifications in native aortic valve leaflets (*arrow*) and in coronary arteries and two pacemaker wires are also visible. **b** CoreValve prosthesis, 6 weeks after implantation showing overgrowth of stent struts with intimal tissue. **c** Ventricular view of same CoreValve obstructed by large vegetations attached to valvar leaflets (*arrow*). Also note the metal probe (*arrowhead*) in small paravalvular leakage. **d** Postmortem X-ray of the heart with Edward Sapien TAVI prosthesis. The stent bearing the artificial valve of this type of TAVI prosthesis is much smaller than of the CoreValve (**a**). **e** Example of mal-positioned Edward Sapien prosthesis, with significant protrusion of native valve leaflet over prosthetic valve leaflets

Based on the findings of our study, we propose a stepwise approach to perform an autopsy after TAVI. Examples of specific findings at autopsy are demonstrated in Fig. 1. Obtaining appropriate clinical information is essential before the heart is removed from the thorax and should include technical aspects of the procedure. Autopsy-related damage, especially at the prosthesis site, should be avoided. Size and weight of the heart provide information on underlying cardiac morbidity. Attention should be paid to the type of prosthesis and the position of the prosthesis in relation to the native valve, and an X-ray could be helpful (Fig. 1). In case of a previous aortic valve replacement, the position of the TAVI prosthesis in relation to the (surgical) prosthetic valve should be assessed. The implantation height and its relation to the ostia of the coronary arteries as well as its relation to the mitral valve should be determined. It is helpful to carefully inspect the valve leaflets for thrombus and vegetation, including sampling for histology and microbial culture. Especially if time has passed between the TAVI procedure and death, it is advised to sample valvar leaflets for features of tissue degeneration. Inspection of in situ valvar (mal) alignment is important for evaluation of paravalvular leakage. Except for cases with large dehiscence, this inspection might be difficult. In patients with late procedural mortality, the valve-bearing stent is encapsulated and the presence of paravalvular leakage should be carefully evaluated with a probe. Thereafter, it is recommended to remove the prosthesis to assess the landing zone in the left ventricular outflow tract, sinus of Valsalva, native aortic valve leaflets and coronary ostia for traumatic, thrombotic, calcific and occlusive pathology. Nitro Blue Tetrazolium (NBT) stain of a fresh myocardial slice at autopsy (to visualise early ischemic areas with LDH depletion) and histology of embedded myocardial tissue provide information not only on the presence of myocardial infarction but also of the time of onset. In a systematic examination of Koch’s triangle, the AV node area is recommended for documentation of potential ischemia and or haemorrhage. With the known high risk of cerebrovascular events during and after TAVI, it is essential to perform cerebral autopsy in all patients after a TAVI procedure.

### Limitations

This study is a retrospective analysis of the autopsy findings from eight different pathology laboratories. Although pathologists with cardiovascular pathology expertise performed all autopsies, there was no standardised protocol. Therefore, autopsy techniques varied to some extent amongst the institutes. Moreover, in the analysed cohort, the number of autopsies after a transapical procedure was relatively overrepresented since transfemoral procedures are usually more common. Furthermore, we analysed 72 cases with procedures from 2007 to 2015. Only a minority of less than one fifth of the procedures was performed before 2010; however, during this wide range in time of the procedure, some technical and procedural aspects for TAVI changed. Newer generation prostheses may show different complications if compared to older prostheses due to changes frame heights and sheath size.

Since this is an elderly patient population, death without autopsy is frequent, especially when it occurs out of hospital. In addition, registries of all laboratories were screened for patients with autopsy and a TAVI in their medical history. If a TAVI was not clearly described in the medical history, these patients were missed in our analysis. Therefore, this observational study does not consist of consecutive cases, and detailed information on autopsy rates amongst the total population of the patients who underwent TAVI and died in the different institutes involved is lacking. Similarly, it is possible that autopsies were requested especially in those cases in which the clinically suspected cause of death was not entirely clear. This may induce an overestimation of the advantage of autopsy over a solely clinically determined cause of death.

## Conclusion

A structured autopsy, performed with knowledge of the general medical history, the technique of the procedure and the time of death in relation to the procedure, reveals valuable information regarding the cause of death after TAVI. In addition to cardiac autopsy, cerebral autopsy is notably of added value. Clinicians should appreciate autopsy over a solely clinically determined cause of death. Pathologists performing those autopsies should have knowledge of the procedure and recognise the importance of the time interval between the TAVI procedure and death.
